# Design principles of bioinspired interfaces for biomedical applications in therapeutics and imaging

**DOI:** 10.3389/fchem.2022.990171

**Published:** 2022-09-23

**Authors:** Chun-Pei Shih, Xiaofang Tang, Chiung Wen Kuo, Di-Yen Chueh, Peilin Chen

**Affiliations:** ^1^ Research Center for Applied Sciences, Academia Sinica, Taipei, Taiwan; ^2^ Institute of Physics, Academia Sinica, Taipei, Taiwan

**Keywords:** bioinspired, interface, nanomaterials, surface modification, drug delivery, nanoparticle (NP), therapeutics

## Abstract

In the past two decades, we have witnessed rapid developments in nanotechnology, especially in biomedical applications such as drug delivery, biosensing, and bioimaging. The most commonly used nanomaterials in biomedical applications are nanoparticles, which serve as carriers for various therapeutic and contrast reagents. Since nanomaterials are in direct contact with biological samples, biocompatibility is one of the most important issues for the fabrication and synthesis of nanomaterials for biomedical applications. To achieve specific recognition of biomolecules for targeted delivery and biomolecular sensing, it is common practice to engineer the surfaces of nanomaterials with recognition moieties. This mini-review summarizes different approaches for engineering the interfaces of nanomaterials to improve their biocompatibility and specific recognition properties. We also focus on design strategies that mimic biological systems such as cell membranes of red blood cells, leukocytes, platelets, cancer cells, and bacteria.

## 1 Introduction

Research on bio-inspired interfaces has gained significant attention in recent years due to their increasing potential in biomedical science. One of the major goals in biomedical research is to improve the efficacy of drugs while reducing side effects, which includes enhancing the delivery efficiency and endowing specific targeting abilities. In addition, biomedical imaging is another important aspect to consider, which provides valuable information on molecular distributions both *in vitro* and *in vivo*. Biomedical applications based on nanoparticles (NPs) have been widely investigated due to their accessibility and versatility. When designing a delivery system, we need to consider the journey of NPs in the body before they reach disease sites, as well as the barriers they will face during their journey. In general, NP-based delivery systems enter circulation through either injection or oral uptake. Therefore, one of the most challenging issues for efficient delivery is reaching the targeted organs while avoiding clearance by the body.

One of the barriers to NPs in circulation is removal by the liver and spleen in the reticuloendothelial system (RES). NPs are deemed foreign substances when administered intravenously and are subjected to sequestration by the liver via Kupffer cells (Tang et al., 2019). Another barrier is the tendency to form protein corona immediately upon entering circulation. When NPs enter a complex biological environment such as the bloodstream, interstitial fluid, or extracellular matrix, they interact with various biomolecules, such as plasma proteins, which trigger corona formation. As proteins start to build up on the surfaces of the NPs, the formation of protein corona signals the mononuclear phagocyte system (MPS) for quick removal (Mirkasymov et al., 2021).

To overcome these barriers, it is essential for NPs to possess the necessary tools to navigate through the body and reach their target site without being removed. One of the common strategies is to mimic the blood cells in circulation by camouflaging the surfaces of NPs with membranes of blood cells, which can prevent clearance by RES and premature expulsion (Xie et al., 2019). It is essential for the designed NPs to spend a suitable amount of time traversing the circulatory system to arrive at their destination (Sushnitha et al., 2020). Another important factor governing the capabilities of NPs is the ability to execute selective targeting—that is, to only interact with the site of interest while leaving other sites intact (cells, tissue, etc.) (Mitchell et al., 2021). Lastly, NPs must be cleared from the body after completing their tasks without inducing any negative impact (Khan et al., 2019; Wang et al., 2021a).

**FIGURE 1:**
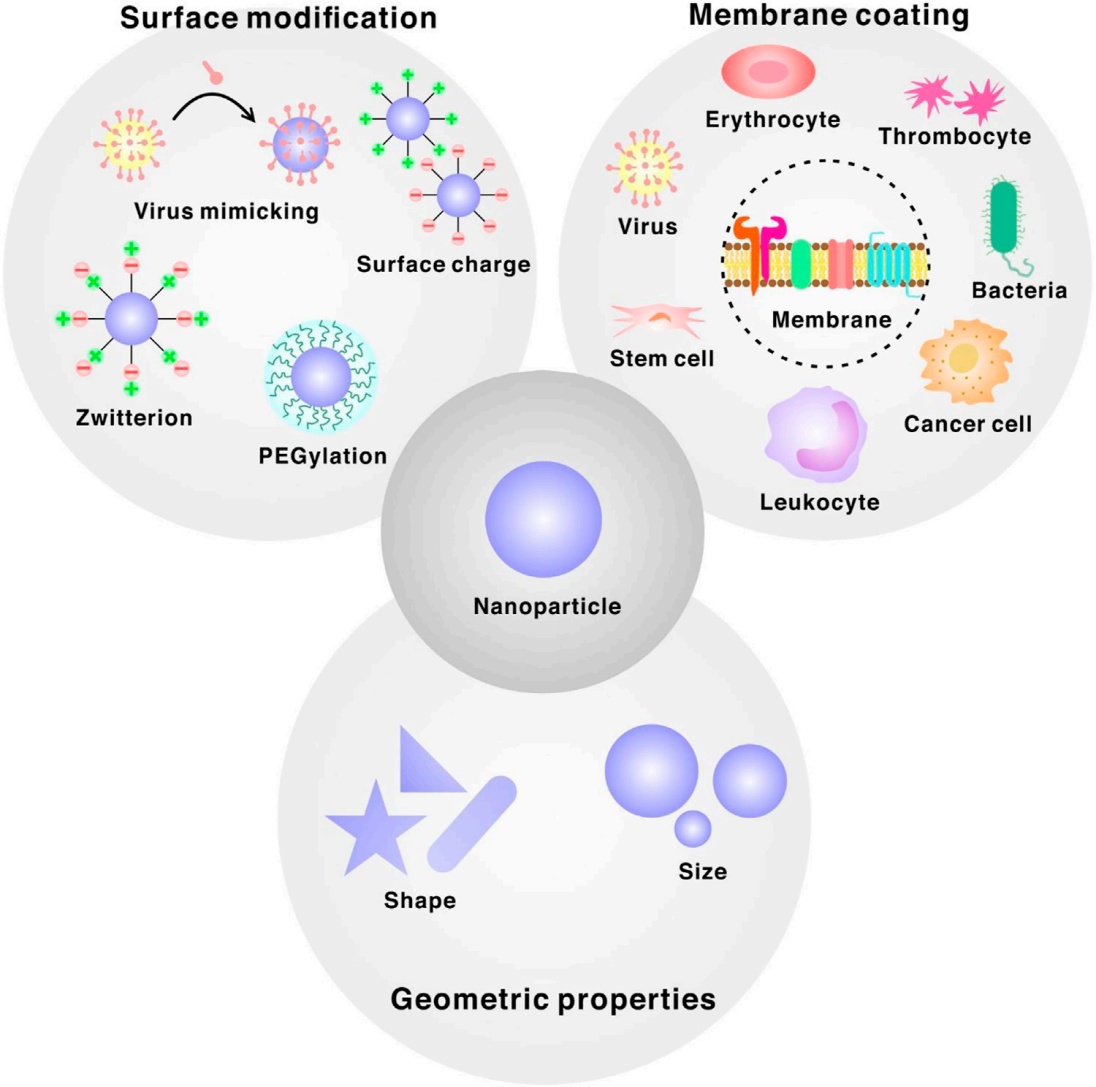
Design principles for bioinspired interfaces of nanoparticle categorized into three different sections. Membrane-coated nanoparticles derived from mammalian cell, cancer cell, bacteria or virus. Surface modification ligands such as PEG, zwitterion, positively/negatively charged ligands or viral capsids. Manipulation of geometric properties of nanoparticles such as size or shape.

These general features are crucial for designing NPs because they ultimately decide the fate of the NPs when administered into the body. The body is a complex biological environment that may vary in complexity in different people. Furthermore, it is challenging for designed NPs to overcome several barriers and retain their intended functions. Therefore, in this mini-review, we present several design principles that may provide insights into using NPs in different situations (Figure 1).

## 2 Design principles for bioinspired interfaces of NPs

**FIGURE 2:**
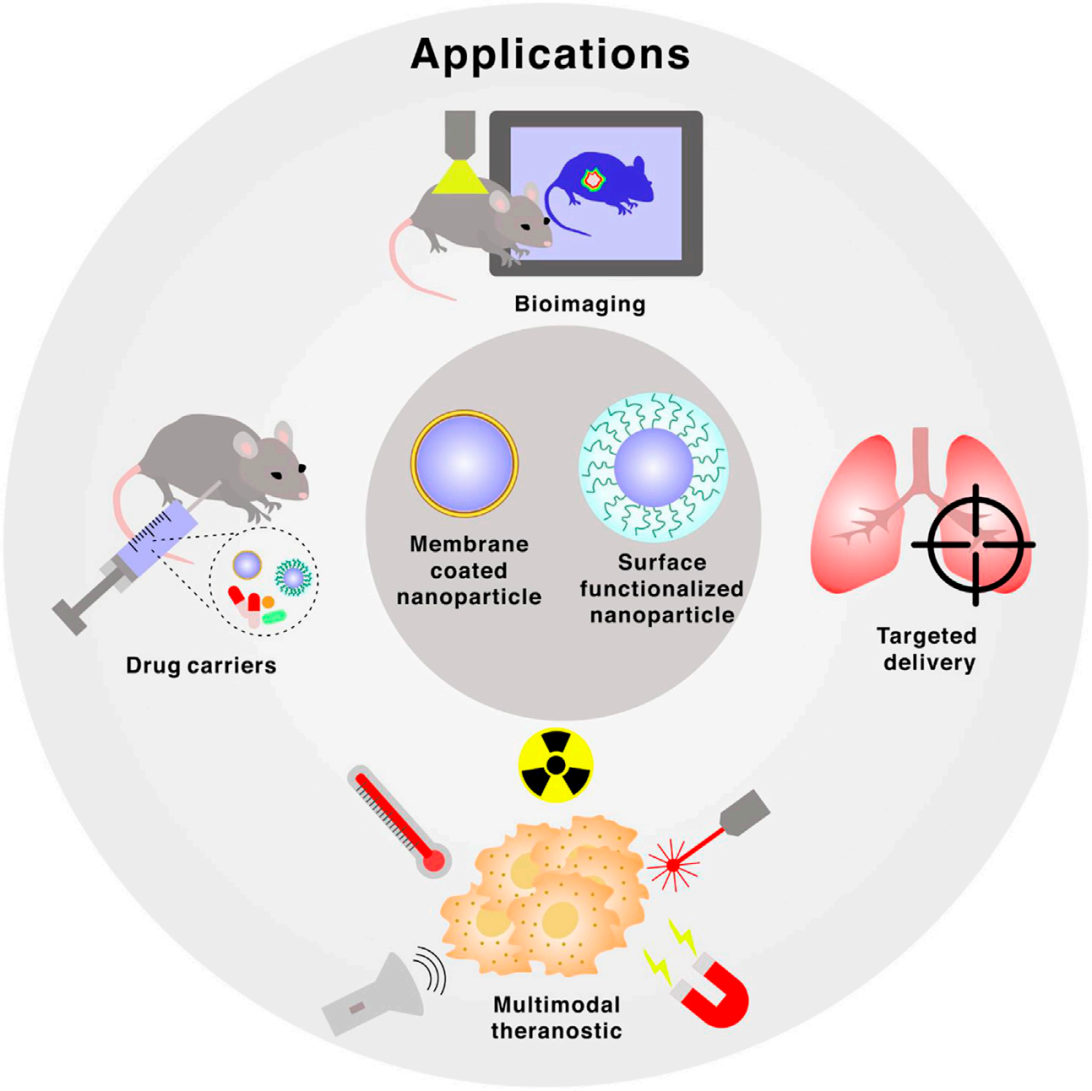
Applications of membrane-coated and surface functionalized nanoparticles, which includes bioimaging, targeted delivery, multimodal theranostic and drug carriers.

NPs have attracted attention among researchers due to the wide array of biomedical science applications, including targeted drug delivery, *in vivo* therapeutics, bioimaging, and cancer treatment (Wang et al., 2020a) (Figure 2). One of the major challenges is to overcome the ability of the human body to recognize and clear foreign materials (Pratiwi et al., 2019; Malachowski and Hassel, 2020). For example, interaction with opsonins triggers quick removal from the body by the MPS (Papini et al., 2020). Furthermore, extensive protein corona formation may also result in the loss of targeting capability of ligand-functionalized NPs, as well as significant changes to NPs’ surface chemistry, thus disabling or altering the desired properties (Rampado et al., 2020).

Without additional surface modifications, NPs can only rely on the enhanced permeability retention effect for entrance into solid tumors, which normally has less efficiency (Subhan et al., 2021). Several strategies have been developed over the years to overcome these problems by either functionalization of NPs or altering their geometric properties. The strategies are categorized in later sections based on the nature of the modifications.

### 2.1 Functionalization of NPs with biomimetic materials derived from biological entities

**FIGURE 3:**
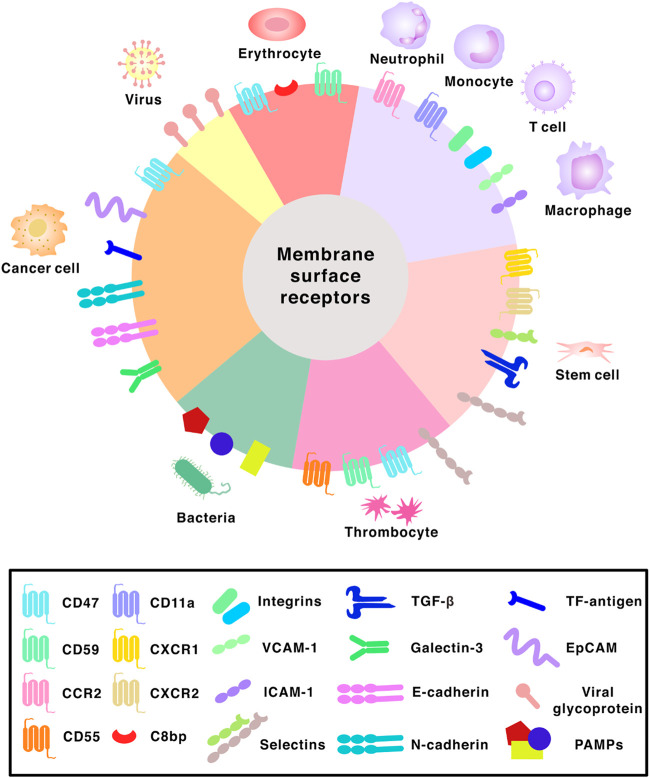
Surface receptors found on different membranes. Abbreviations: CCR2, C-C chemokine receptor 2; CXCR1, C-X-C chemokine receptor 1; CXCR2, C-X-C chemokine receptor 2; C8bp, C8 binding protein; VCAM-1, vascular cell adhesion molecule-1; ICAM-1, intercellular adhesion molecule-1; TGF-β, transforming growth factor beta; TF-antigen, Thomsen-Friedenreich antigen; EpCAM, epithelial cell adhesion molecule; PAMPs, pathogen associated molecular patterns.

Biomimetic materials can reproduce or recapitulate the properties of their biological counterparts in terms of chemistry, structure, characteristics, and functions (Perera and Coppens, 2019). Red and white blood cells possess the ability to circulate freely in the bloodstream without being expelled by the MPS. Platelets are able to evade phagocyte uptake and contain surface receptors that can target specific sites for tissue repair during an injury. Other cells such as cancer cells or stem cells have also been investigated (Figure 3). Hence, the concept of membrane-coated NPs (MNPs) has arisen and has been utilized in NPs to improve their circulation half-time after administration into the bloodstream, perform drug delivery to a specific site in the human body or use as contrast agents for bioimaging purposes. (Zou et al., 2020).

In addition to blood cells, studies have also been done on pathogen-based materials derived from bacteria (Jiménez-Jiménez et al., 2020) and viruses (Park et al., 2022), which exhibit some unique properties in delivering payloads. Therefore, it is important to understand the characteristics of different biological entities and exploit their functions for therapeutic applications. In this section, we discuss how researchers are taking advantage of the unique properties of these natural biological materials in designing NPs for biomedical applications and the challenges that they may face.

#### 2.1.1 Erythrocyte membrane

Red blood cells (RBCs) are the most commonly found cells in the human body. The properties of RBCs include biocompatibility, prolonged circulation time, and biodegradability, which make them a perfect candidate for efficient NP carriers. The CD47 “marker-of-self” proteins found on RBC surfaces are able to inhibit phagocytosis of RBCs by immune cells, thus preventing degradation and improving the circulation time significantly (Guo et al., 2021). This feature has been incorporated into many drug-delivery systems by coating NPs with RBC membranes (RBCMs) (RBCM-NPs).

The earliest successful fabrication of RMCM-NPs was achieved by Hu et al., where they encased poly (lactic-co-glycolic acid) (PLGA) NPs with RBCM. The CD47 proteins were retained with the correct orientation, thus allowing the encased NPs to reduce macrophage engulfment by 64%. It was also shown that RBCM-NPs have a longer elimination half-life than other surface-modified NPs (Hu et al., 2011; Hu et al., 2013). In another example, RBCM-coated PLGA NPs loaded with rapamycin demonstrated high targeting specificity towards atherosclerotic plaques, where the development of the disease was delayed significantly, and prolonged administration did not cause serious side effects in the mice model (Wang Y. et al., 2019a).

Other than disease treatment, RBCM-NPs were also employed in many cancer-related studies. RBCM-coated PLGA NPs loaded with gambogic acid display enhanced anticancer effects both *in vitro* and *in vivo* (Zhang L. et al., 2017a). In another study, RBCM-coated mesoporous silica nanoparticles containing doxorubicin successfully prevented breast cancer metastasis (Su et al., 2017). Although many designs of RBCM-NP have shown promising results in cancer treatment, RBCM lacks the natural tumor-targeting ability and can only rely on the enhanced permeability and retention (EPR) effect (Xia et al., 2019).

In order to improve the targeting ability of RBCM-NP, decorating ligands were added to the RBCM via chemical synthesis (Wu et al., 2019). It was demonstrated that the cancer-targeting ability was enhanced significantly when epithelial cell adhesion molecules were added to the surface of RBCM-coated gold nanocage (Zhu et al., 2018). Other than chemical synthesis, the lipid insertion method also enables the targeting functionalization of RBCM-NPs (Fang et al., 2013).

The superiority of RBCM-NPs not only excels in the area of drug therapeutics but also in the area of bioimaging, more specifically for *in vivo* imaging of tumors. Upconversion NPs (UCNPs) were commonly used for *in vivo* fluorescence imaging but suffered from diminished targeting capabilities when administered into the body due to protein corona formation. To resolve this issue, RBCM was used to eliminate the tendency of protein corona formation in UCNPs. Further addition of cancer-targeting molecules onto the RBCM surface resulted in RBCM-coated UCNPs with excellent optical capabilities that show enhanced accumulation at tumor sites (Rao et al., 2017). The vast development in dual-functionalized RBCM-NPs was also yielding impressive results in bioimaging. RBCM-coated semiconducting polymer NPs (RBCM-SPNPs) with both photoacoustic imaging (PAI) and photothermal therapeutic (PTT) properties were developed. Results have shown that the RBCM-SPNPs significantly improved PAI signals and PTT capabilities (Zheng et al., 2020).

#### 2.1.2 Leukocyte membrane

Also known as leukocytes, white blood cells (WBCs) are immune cells protecting the body against threats such as infections, foreign invaders, and pathogens (Glenn and Armstrong, 2019). The diverse and versatile functions of WBCs have promoted the development of WBC membrane-coated NPs due to their ability of cellular self-recognition, crossing biological barriers of the body, and preference to bind to receptors at disease sites (Harjunpaa et al., 2019). By taking advantage of such characteristics, WBC membrane-coated nanoporous silica particles were developed and could retain WBC traits such as avoiding clearance by the immune system and traversing the endothelial layer (Parodi et al., 2013; Gong et al., 2020).

Various WBCs are frequently employed as NP carriers, such as neutrophils, macrophages, monocytes, and T cells. For example, neutrophil membrane-coated NPs successfully targeted and reduced bacterial load at bacteria-infected sites in mice by mimicking neutrophils’ characteristics to interact with inflamed tissues or cells via adhesion proteins (Zhang C. et al., 2019a). Macrophages have also been extensively studied due to their specific targeting ability for inflammation and tumor endothelium. This capability was demonstrated in macrophage membrane-coated NPs (MM-NPs) when they were used to inhibit the progression of atherosclerosis in mice. It was shown that the treatment regime was able to effectively target and accumulate atherosclerotic lesions *in vivo* (Wang et al., 2021a). The use of MM-NPs in cancer treatment and bioimaging was also very active. In a study, a persistent luminescence NP-derived nanocomposite containing the drug was coated with a macrophage membrane. The macrophage membrane-coated nanocomposite has shown great potential in autofluorescence-free imaging as well as enhanced tumor inhibition capabilities (Chen et al., 2020). As promising as MM-NPs sound, however, studies have pointed out limitations such as the ability only to target limited types of tumors (Liang et al., 2020; Sun et al., 2020).

Monocyte recruitment occurs as a natural response when there is a change in the physiological environment in the body. Specifically, the ability to infiltrate different sites of interest varies (Kratofil et al., 2017; Lehmann et al., 2017; Teh et al., 2019)). Hence, by exploiting the ability of monocytes, a lipid NP-based drug-delivery platform was designed specifically to attach to the surface of circulating monocytes. The monocytes would then carry and extravasate with the lipid NPs at the diseased site (Huang et al., 2021).

It was mentioned earlier that the membranes of WBCs contain various proteins on the surfaces to detect inflammation and diseased tissues. T cells possess a higher level of targeting proteins than general WBCs and more easily accumulate at a tumor site. Therefore, the membranes of T cells were also employed as a camouflaging mechanism for NPs to prolong circulation by avoiding phagocytosis while taking advantage of the surface adhesion molecules for cancer targeting (Zhang Z. et al., 2017b). For bioimaging application, azide-modified T cell membrane coated NPs were shown to have excellent fluorescence intensity signal as well as enhanced photothermal response (Han et al., 2019). In several studies, it was revealed that there is a reliance on the activation of T-cell receptor-peptide-major histocompatibility complex interaction to eliminate cancer cells. As effective as it may seem, the interaction was not effective against solid tumor treatment due to the deficiency of tumor-specific biomarker presented by solid tumor cells (He et al., 2019).

#### 2.1.3 Thrombocyte membrane

Platelets are a component in blood that are responsible for initiating a blood clot during blood vessel injury. Compared to other forms of membranes for NP coating, platelet membranes are advantageous due to their ability to promote drug targeting both actively and passively. Similar to RBC membranes, platelet membranes also possess CD47 “marker-of-self” proteins that prevent immune clearance (Olsson et al., 2005; Han et al., 2022). Hence, the circulation of platelet membrane-coated nanoparticles (PM-NPs) can be prolonged, achieving passive drug targeting.

Platelet membranes also contain a set of unique surface receptors such as glycoprotein Ib that can bind to the exposed collagens of the damaged vasculatures to trigger tissue repair or bind directly to pathogenic bacteria, causing platelet aggregation (Deppermann and Kubes, 2018). These surface receptors are passed onto PM-NPs, thus enabling active drug targeting (Hu et al., 2015). Due to these characteristics, platelet membranes have become a popular choice in the development of nanotherapeutics (Wang S. et al., 2020c). Platelet-like proteoliposomes were fabricated using platelet membranes that could interact strongly with circulating monocytes, which could significantly improve post-infarction therapy (Cheng et al., 2016).

PM-NPs could also be used as an antibody decoy for the treatment of immune thrombocytopenia as they possess native platelet surface proteins that can strongly interact with anti-platelet antibodies (Wei et al., 2016). Another common application of PM-NPs is in the area of cancer cell detection, drug-delivery treatment, and tumor imaging by exploiting the specific interaction between platelets and various cancer cells. Docetaxel-loaded PLGA NPs coated with platelet membranes were compared with free docetaxel and an uncoated control by analyzing the biodistribution of the drug *in vivo* in a mouse model of human lung cancer. The results showed that docetaxel-loaded PLGA NPs coated with platelet membranes were able to retain the highest concentration of drugs in tumors, thus achieving the highest inhibition of tumor growth *in vivo* (Chi et al., 2019). Other than docetaxel, PM-NPs were also used to deliver other anti-cancer drugs to treat various cancers (Shang et al., 2019; Xu et al., 2019). A PM-NP-derived second near-infrared window (NIR-II) phototheranostic nanoprobes were developed for aggressive active targeting of various types of cancer. From the results, the nanoprobes displayed outstanding photoacoustic imaging ability and enhanced photothermal conversion efficiency (Geng et al., 2020).

Even though PM-NPs possess advantages that succeed as an excellent candidate for biomedical application, it was shown that patients with autoimmune diseases may develop platelet autoantibodies, which form immune complexes with PM-NPs triggering removal by the immune system (Nelson et al., 2021). Hence, further studies should be conducted to provide more comprehensive and risk-free development of PM-NPs.

#### 2.1.4 Cancer cell membrane

The development and progression of cancer involve a multi-stage process in which cancer cell adhesion molecules (CCAMs) play an important role in metastasis (Okegawa et al., 2004; Bendas and Borsig, 2012). CCAMs have various surface receptors that are crucial for intravascular heterotypic or homotypic adhesive interaction, which ultimately leads to the build-up of metastatic deposits (Sun et al., 2016; Harjunpaa et al., 2019). CD47 surface proteins are also expressed on the surfaces of cancer cells, which provide them with the ability to avoid the immune system (Lian et al., 2019; Huang C.-Y. et al., 2020a). Taking advantage of these characteristics, cancer cell membranes (CCMs) have been used to coat NPs.

CCM-coated NPs (CCM-NPs) possess the ability to evade immune detection as well as the capability to target homologous cancerous sites or tumors. CCM-NPs could also be tuned specifically to cater to specific needs of cancer treatment (Li et al., 2017). The homotypic targeting ability of CCM-NPs was investigated by CCM derived from the same type of cancer cells. As compared to uncoated NPs and RBCM-NPs, the CCM-NPs display a strong homotypic affinity to the source cancer cells, which led to significantly higher cellular uptake (Fang et al., 2014). In another example, it was demonstrated that by adjusting the source of the cell membrane coating of CCMNPs, it can achieve self-recognition internalization by the source cancer cell lines as well as highly selective targeting to the homologous tumor *in vivo* when homotypic CCM was used (Zhu et al., 2016). From the previous example, personalization of cancer treatment can be advantageous as drug delivery to the site of cancer metastasis was significantly enhanced. The feasibility of this concept was further proved by using NPs with a programmed CCM that could target the bone specifically and enhance homotypic tumor uptake (Gdowski et al., 2019).

Owing to the homologous targeting ability, CCM-NPs were commonly employed in tumor imaging and were often functionalized with more than just imaging capabilities (Rao et al., 2016; Li J. et al., 2018a). Most recently, novel iridium complexes functionalized black-titanium NPs coated with CCM were developed. The NPs possess a hierarchical dual targeting ability, which can selectively reside in the mitochondria and exhibit enhanced accumulation in cancerous cells. It can also generate efficient photothermal capability upon NIR-II irradiation and generate reactive oxygen species upon ultrasound radiation. The combination of two stimulating factors produces a high-resolution image of the tumor site and triggers the eradication of tumor cells in mouse models (Shen et al., 2021).

As the development of CCM-NPs progresses, safety concerns should be evaluated thoroughly. For instance, during the preparation of CCM-NPs, one should ensure the complete removal of nuclear and genetic material within the cancer cell, which may possess carcinogenic risks (Xu et al., 2020).

#### 2.1.5 Stem cell membrane

The most widely studied and used type of stem cells in the field of biomedical research are mesenchymal stem cells (MSCs) because of their easy isolation and tumor-targeting properties (Zhao W. et al., 2017a; Aravindhan et al., 2021). Applications of MSCs for NP-based drug delivery systems have been demonstrated by several research groups. For instance, different types of NPs were loaded into MSCs, and their effects were investigated. It was shown that loaded NPs were successfully internalized into MSCs without affecting cell viability and differentiation. Most importantly, it was also demonstrated that the designed MSC membrane-coated NPs (MSCM-NPs) displayed high selectivity toward an experimental human glioma model (Roger et al., 2010; Wang et al., 2018).

MSCM-NPs were employed in numerous cancer-related studies, for instance, MSC membrane-coated gelatin nanogel was designed to have high tumor affinity. Due to the variety of molecular recognition moieties on the MSC membrane, the results demonstrated high stability and high selectivity toward tumors both *in vitro* and *in vivo* (Gao et al., 2016). In another study, PLGA NPs coated with MSC membrane displayed high anti-tumor efficiency in an orthotopic breast cancer model (Tian et al., 2019). Taking advantage of highly specific tumor homing ability, MSCM-NPs were also frequently used for bioimaging applications (Lai et al., 2015; Mu et al., 2018). More recently, Chetty et al. developed a novel, biocompatible MSCM-NP with multimodal imaging capabilities that serve as an outstanding imaging agent in near-infrared fluorescence, magnetic resonance, and computed tomography (Chetty et al., 2020).

Although MSCM-NPs have shown excellent tumor-targeting capabilities, it should also be noted that there are controversies over whether MSCs promote tumor growth (Javan et al., 2019) or inhibit it (Cheng et al., 2019). More evidence is needed to highlight the advantages and disadvantages of MSCs when used in cell-mediated gene strategies (de la Torre et al., 2020).

#### 2.1.6 Bacterial membrane

Bacteria are ubiquitous single-celled organisms that are important to the ecosystem. Some bacteria, however, are pathogenic and may cause infectious diseases. The mechanism of how they evade the immune system and trigger favorable interaction with target cells is a subject of interest due to its potential applications in therapeutics (Yoo et al., 2011; Sushnitha et al., 2020). Bacterial membranes contain many immunogenic antigens and adjuvants that are responsible for innate immunity stimulation and adaptive immune response promotion (Poetsch and Wdlters, 2008; Chen B.-M. et al., 2021a; Prior et al., 2021). It is strategic to coat bacterial membranes onto NPs because not only do the characteristics of both NPs and bacteria remain intact, but they can also mimic the natural antigen interactions between bacteria and the immune system (Gao et al., 2015; Anwar et al., 2021).

Generally, bacterial membrane-coated NPs (BM-NPs) are fabricated by coating NPs with processed bacterial outer membrane vesicles (OMVs). Naskar et al. discuss the details of the preparation of OMVs and fabrication of BMNPs (Naskar et al., 2021). BMNPs are relatively new in the field of cell membrane-coated NPs, and comprehensive research studies are still under development. Nevertheless, BM-NPs have several unique advantages that outshine other membrane-coated NPs.

It was demonstrated that PLGA NPs coated with OMVs originating from *S. aureus* can actively target macrophages infected with *S. aureus in vitro,* as well as major organs bearing metastatic infections in *S. aureus* bacteremia-bearing mouse models. Compared to the *E. coli* OMV control, the *S. aureus* BM-NPs exhibit excellent selectivity towards *S. aureus*-infected macrophages and organs. The selectivity of the BMNP became specific to *E. coli* when the origin of the OMV used was switched from *S. aureus* to *E. coli*, demonstrating the unique advantage of using bacterial membranes (Gao et al., 2019). This advantage of bacteria-specific targeting was not shown in other kinds of membrane-coated NPs.

BM-NPs were also used in the development of anti-bacterial vaccines to provide another alternative to antibiotics due to the rapid emergence of bacterial drug resistance (Naskar and Kim, 2019). As mentioned earlier, bacterial membranes contain various immunogenic antigens with intrinsic adjuvant properties. The integration of synthetic NPs and bacterial membranes resulted in BM-NPs that retain the biological characteristics of bacteria as well as the physicochemical properties of the synthetic NPs. A BM-NP was developed by coating size-controlled bovine serum albumin NPs with OMVs from carbapenem-resistant *Klebsiella* pneumonia (CRKP). These BM-NPs were used to provide immunization to mouse models, which endowed them with an increase in survival rate when infected with a lethal dose of CRKP (Wu et al., 2020).

Certain species of bacteria have natural tumor targeting properties and were applied in cancer therapeutic (Van Dessel et al., 2015; Jiménez-Jiménez et al., 2022). The discovery of the presence of bacteria in human tumors has also provided insights regarding the usage of BM-NPs for cancer therapy or tumor imaging (Nejman et al., 2020). A cancer vaccine based on a polyplex core containing adjuvants coated with imide groups and the bacterial membrane was developed. When combined with radiotherapy, immunogenicity generated by the designed BM-NPs exhibits enhanced inhibition of tumor growth and produces anti-cancer immune memory (Patel et al., 2019). The applications of BM-NPs in bioimaging were also demonstrated in several studies (Wang Z-. H. et al., 2019b; Zhang Y. et al., 2019b).

The use of bacterial membranes as a coating agent for NPs is still an ongoing research topic that requires thorough studies in order to overcome some issues raised by researchers. It was shown that the size of OMVs has a distinct effect on the mechanism of the entrance to host cells (Turner et al., 2018). Studies of the cytotoxicity of BM-NPs are needed for practical biomedical applications to be possible. This problem was partially resolved in work where lipopolysaccharide neutralizing peptides were used to reduce the inflammation response of BM-NPs. However, this only addresses the problem on a cell-type-specific level instead of a universal level (Pfalzfraff et al., 2019). These issues need to be resolved so that BM-NP-based vaccines and therapeutics can undergo further improvement.

#### 2.1.7 Virus-derived strategies

Viruses have been called nucleic acid carriers due to their ability to protect and deliver a segment of nucleic acid that it encases within its protein outer shell. The ability to evade immune system recognition and transfer genes into host cells for self-replication is exploited in the development of biomedical applications (Elzoghby et al., 2012). Initial development has involved the use of viral gene vectors such as adenoviruses or retroviruses to deliver specific genes of interest into host cells. However, the pathogenic nature raises concerns regarding safety and unwanted immunogenicity, potential toxicity, and mutagenesis. In addition, the limitation of size and cargo hold capacity decreases the versatility of using viral gene vectors (Yoo et al., 2011; Park et al., 2019).

As alternatives, virus-like particles (VLPs) and virosomes were introduced. VLPs are self-assembled particles that mimic the capsid structures or envelope proteins that originate from real viruses (Nooraei et al., 2021). Virosomes are liposome-like particles that contain integrated surface glycoproteins but not the capsid proteins with real virus origin (Asadi and Gholami 2021). Both types of particles do not contain the genetic materials of viruses and form hollow structures that can encapsulate a wide array of payloads (Parodi et al., 2017). The typical characteristics of viruses are retained in these derived entities, including cellular entry, immune evasion, and specific targeting. Thus, they offer promise for the development of drug delivery, imaging, immunotherapy, and theranostic applications (Kuo et al., 2011; Chung et al., 2020).

Similar to other NP functionalization strategies that involve the coating of NPs with cell/bacterial membranes, the encapsulation of NPs within a viral coating protein is also possible. A genetically engineered hepatitis B core VLP was used to encapsulate magnetic NPs with high efficiency and showed potential in the application of magnetic resonance imaging due to enhanced cellular uptake of the VLP-coated NPs (Shen et al., 2015). Not all virus-derived strategies involve the coating of NPs with viral-derived entities. Metallic NPs were coupled to a targeted adenoviral (Ad) platform, and the NP-labelled Ad vector did not have lower infectivity and tumor-targeting capability than the unlabeled control, thus providing another alternative for targeted delivery of NPs (Saini et al., 2008). Other virus-derived strategies mostly involve surface modifications of NPs to mimic that of a virus, which will be discussed separately.

#### 2.1.8 Challenges for biological entities derived functionalization

We have discussed several biological entities derived functionalization of NPs, most of which involve the encapsulation of NPs with different cell membranes to endow functions such as immune system evasion, specific targeting, or bypassing certain biological barriers (Liu et al., 2019). Although the benefits brought forth by the technique are promising, the difficulties behind the fabrication process should be carefully evaluated. The protocols for fusion between cell membrane vesicles and NP cores have yet to be standardized, different protocols stand on their own with varying advantages and disadvantages. For instance, fusion methods such as extrusion can produce uniform size particles but the complexity and time required for preparation cause difficulties in mass production (Xu et al., 2020).

In addition, the cell membrane coating integrity was found to affect the internalization pathway, and most of the proposed methodology currently produces a non-homogeneous mixture of uncoated, partially coated, and fully coated NPs. The solution to separate fully coated NPs from the non-homogenous mixture is critical for future improvement (Liu et al., 2021a). The capabilities of different MNPs must undergo a more comprehensive study to better classify each of them according to their therapeutic effect (Lei et al., 2022).

The future for MNP fabrication should focus on refining current methodologies while aiming toward the development of a highly efficient and effective universal protocol for cell membrane extraction as well as fusing cell membrane vesicles and NP cores. The automation of these processes is crucial for initial development as this marks the first step toward industrial-level production. Overall, the future development for MNP should focus more on process development instead of discovery (Li R. et al., 2018b).

### 2.2 Functionalization of NPs via surface modification techniques

The surface modification of NPs is an important aspect of designing NPs to achieve specific biomedical functions. It is an effective yet simple way to easily change the characteristics of NPs. For example, conjugation of poly (ethylene glycol) (PEG) onto the surfaces of NPs may allow them to evade MPS clearance. Conjugation of functional groups onto the surfaces of NPs can be fine-tuned to alter the surface electrical charges, which can affect the rate of cellular uptake. There are also strategies to mimic viruses to endow NPs with viral-like properties. This section summarizes the typical ligands used for surface modifications, as well as their advantages and disadvantages.

#### 2.2.1 PEGylation

The concept of PEGylation of polymeric NPs was first described in 1977, when PEG molecules were attached to NPs in an effort to increase their circulation time in the bloodstream (Gref et al., 1994). The significant improvement in the circulation time of NPs was due to the decrease in the occurrence of aggregation, opsonization, and phagocytosis. These phenomena are responsible for the accelerated clearance of therapeutic NPs from the body via MPS. However, when the PEG chains are grafted onto the surfaces of NPs, a hydrophilic brush layer forms, thus endowing the NPs with an ‘anti-fouling’ surface. Since the PEG-grafted NPs were shielded from nearby NPs and blood components that may trigger MPS recognition, they gained a prolonged circulation half-life as a result (Shi L. et al., 2021a; Mitchell et al., 2021).

The advantages of PEG prompted advancements in therapeutic NPs. A variety of PEG-decorated NPs with different applications has emerged over the years. For instance, PEGylated MSNs loaded with different pH-sensitive dyes were used to investigate the intracellular pathway of NPs (Zhang et al., 2020). PEGylated NPs have been widely employed in studies of cancer treatment, such as sonodynamic therapy (Wang et al., 2020b) and tumor targeting (Liu et al., 2021b). PEGylation of NPs also enables enhanced stability and increased biocompatibility. This was demonstrated when PEGylated chitosan-modified gold NPs exhibited colloidal stability in a complex biological environment (Liu et al., 2015). More examples of nanodrug can be found elsewhere (Patra et al., 2018).

Currently, the most important application of PEG-decorated NPs is in the area of vaccine development for COVID-19. Briefly, mRNA-based vaccines are fabricated using lipid NPs decorated with PEG on the surfaces for efficient mRNA transfection (Pardi et al., 2018; Corbett et al., 2020). PEGylation of NPs can also be seen frequently in the applications of imaging and therapy (Jokerst et al., 2011). There is a long list of benefits that are brought forth by PEG, but there are reports that several drawbacks are caused by PEG polymers. Undesired immunological response involving specific and nonspecific recognition by the immune system often leads to hypersensitivity reactions. The exact mechanism involved has yet to be determined, and there is no exact conclusion about whether PEG alone or a series of combined reactions is the culprit (Knop et al., 2010).

There is also evidence showing that anti-PEG titers can occur in some patients or those immune responses may develop after administering multiple doses of PEGylated NPs (Kozma et al., 2020; Chen L. et al., 2021b). Nevertheless, even with the drawbacks, PEGylation will continue to strive in the area of nanomedicine research due to its excellent benefits, including reduced immunogenicity, antigenicity, and toxicity. Future researchers should be aware of the negative impacts so that more comprehensive studies can be completed (Shi Y. et al., 2021b).

#### 2.2.2 Zwitterions

With rising concerns regarding the disadvantages of PEG, many research groups are seeking alternatives. Many have turned to other synthetic polymers such as biodegradable poly (glutamic acid) (Li and Wallace, 2008; Hoang Thi et al., 2020) or non-biodegradable polymers with close structural similarity to PEG, such as poly (glycerol) (Qi and Chilkoti, 2015). In this search, zwitterionic materials have attracted many researchers’ attention. Similar to PEG, zwitterionic materials are able to extend the blood circulation half-life of NPs without triggering the immune response (Pombo-Garcia et al., 2016). Zwitterionic materials are known to simultaneously possess cationic and anionic moieties in equal proportion, which enables overall charge neutrality and superhydrophilicity (Shao and Jiang, 2014; Zhang et al., 2022).

Zwitterionic materials have also shown strong resistance to nonspecific protein adsorption due to the strong hydration layer formed via strong electrostatic interaction (Jiang and Cao, 2010). However, this property can cause hindrance during interaction with target cells, lowering cellular uptake efficiency (Muro et al., 2010). This disadvantage could be resolved by modifications via the coupling of various special functional groups (Zhang et al., 2010; Debayle et al., 2019). Therefore, the use of zwitterionic material as surface grafting agents can effectively enhance the biocompatibility and stability of NPs for prolonging circulation time *in vivo* while providing a highly tunable interface for different applications.

By exploiting the flexibility of zwitterions, a ratiometric pH sensor based on quantum dots with high stability was developed to map the intercellular pH difference during endocytosis (Pratiwi et al., 2016). In another work, a pH-sensitive zwitterionic material was used to envelop NPs, which enabled them to traverse the bloodstream without MPS detection due to the neutral surface. When the NPs reach a tumor site, the acidic microenvironment of the tumor triggers the exposure of positively charged functional groups on the surfaces of the NPs, which effectively enhances cellular uptake and accumulation (Ou et al., 2018). A similar demonstration of a different pH-sensitive zwitterion material was also shown in another work (Piao et al., 2018).

Other than pH sensitivity, zwitterionic materials can be functionalized to obtain reduction-responsive properties. The intracellular components of tumors typically overexpress glutathione, which can be used to promote and increase the intercellular drug-release rate (Zhao et al., 2017b). By modifying zwitterionic material conjugated on NPs with disulfide linkers, a reduction-sensitive NP delivery platform was developed (Zhu et al., 2015). A dual-sensitive zwitterionic drug-delivery system composed of both pH and redox-sensitive functional groups is also possible (Cai et al., 2015). The vast applications of zwitterionic material conjugates can allow them to serve as an alternative for surface modifications. Other stimulating factors such as temperature or light can also be incorporated into zwitterionic materials for different applications (Zhou et al., 2020). A multifunctional zwitterion decorated NP displayed excellent properties for imaging-guided cancer therapy. When tested with a tumor mouse model, the results have shown enhanced efficiency in tumor destruction as well as increased contrast during magnetic resonance imaging (Zheng et al., 2019).

The use of zwitterionic material for NP coating has shown increasing popularity for different biomedical applications (Peng et al., 2020; Ren et al., 2020; Runser et al., 2020). As the area for zwitterionic material application expands, more problems were being exposed. The higher tendency to form protein corona for some species of zwitterionic material may affect its functionalization (Debayle et al., 2019). The development has yet to reach the stage for real-life applications due to the lack of comprehensive studies. For future improvement, it will require the joint effort of different professionals from various fields of study (Harijan and Singh, 2022).

#### 2.2.3 Surface electrical charge

Surface charge affects the cellular uptake and fates of NPs (Sabourian et al., 2020). When designing the interfaces of NPs, one should be aware of the surface charges of the targets. Biomedical applications of NPs normally target different cells for specific drug deliveries. Normally, cell membranes have a negative charge, which is a natural target for positively charged NPs (Jo et al., 2015; Nishino et al., 2020). Negatively charged NPs are expected to experience diminished cellular uptake.

When NPs are administered to the body, they are subjected to a complex microenvironment where various proteins reside. This increases the chance of adsorption of different proteins onto the surfaces of NPs, which results in the formation of a protein corona. The hydrophobic aggregation of various proteins on the NP surfaces may alter their functionality and biological nature, causing them to lose their intended function or gain unwanted properties (Tenzer et al., 2013; Park, 2020). The chances of non-specific internalization of NP may increase when different proteins are adsorbed onto the surfaces of NPs, changing the surface charges or the properties of the NP completely. Therefore, the design of the NPs ultimately is decided by whether one wants to avoid unwanted protein adsorption or to take advantage of the protein adsorption.

The surface charge effect of NPs can be studied by tuning the surface charges of NPs through the conjugation of functional groups onto the surfaces (Du et al., 2018) and by subjecting the NPs to different microenvironments (Lesniak et al., 2012). In summary, the overall surface charge as well as the charge density of NPs should be examined closely for both *in vitro* and *in vivo* experiments in order to elucidate the effect of surface charges on NPs. In addition, a better understanding of how corona formation affects the surface charge of NPs will help the design of NPs (Albanese et al., 2012; González-García et al., 2022).

#### 2.2.4 Virus mimicking

This section discusses surface modifications of NPs to mimic various features and properties of viruses. It was observed that the surface topology of a virus greatly affects the interactions between host cells (Dimitrov, 2004; Huang Y. et al., 2020b; Pizzato et al., 2022). Therefore, one of the strategies is to mimic the surface topology of enveloped viruses. For instance, adding smaller silica NPs onto larger silica NPs increases the surface roughness of the large silica particles. This augmentation significantly promotes the interactions between the silica NPs and target cells, thereby enhancing delivery efficiency (Niu et al., 2013).

Other than surface topology, mimicry of viral architecture is another strategy that many research groups have investigated. The viral capsid is a subject of interest related to mimicking viral architecture since it is crucial for cellular targeting and entry. Unlike natural virus vectors such as VLPs or virosomes, viral capsids can be fabricated with synthetic building blocks that possess specific targeting functions, which their alternatives lack (Matsuura, 2018; Aljabali et al., 2021). In the case of tumor targeting, a multifunctional viral mimic, fabricated from self-assembled amphiphilic dendritic lipopeptides was shown to possess virus-like infection capabilities for solid tumor and tumor cells. By taking the advantage of typical properties and characteristics of viruses and additional tuning of the viral mimic, tumor suppression was achieved in testing *in vitro* and *in vivo* in comparison to the control groups (Zhang et al., 2015). Viral mimics were also applied for increased therapeutic efficiency in antimicrobial-related studies (Shi L. et al., 2021a) as well as biomedical applications in cancer research (Gao et al., 2021).

Viral capsid mimicking can also be function-specific by endowing the viral capsid with stimuli-responsive receptors and enabling interactions with a targeted site. In this way, NPs gain the ability to disassemble and deliver their cargos, which is advantageous for intracellular interactions (Xu et al., 2014; Chen et al., 2019). Surface modification techniques involving virus mimicking can be beneficial as they completely eliminate the use of viral components that are essential for infection inducement in VLPs and virosomes. It can be anticipated that future research in this area will work towards the development of multifunctional artificial “viruses” that overcome several limitations of current NP drug-delivery systems (Parodi et al., 2017; Figueroa et al., 2021).

#### 2.2.5 Challenges for surface modification techniques

Surface modification techniques offer a wide variety of functionalization for NPs, the use of decorating ligands or molecules was even observed for some NPs to provide additional features (Zhu et al., 2018; Wu et al., 2019). Owing to the high variability of these decorating materials for NP surface modification, many challenges arise. Firstly, one must consider the conjugation density and orientation of ligands on the surface of NP as these factors can affect cellular uptake efficiency. Secondly, there is a lack of standardized analytical methodology for a comprehensive evaluation of NP with different surface modifications (Takechi-Haraya et al., 2022). Lastly, the change of geometric properties of NP must be taken into consideration (Sanità et al., 2020), because these changes may also influence cellular uptake or even result in a complete loss of the intended functionalization. More details regarding the effect of geometric properties of NP will be discussed in the next section.

### 2.3 Functionalization of NP with geometric property variations

Previous sections have discussed NPs subjected to protection or transportation via membrane coatings, which prolong the NPs’ half-life during blood circulation or act as a vehicle to carry NPs to specific sites of interest. We have also seen various surface-modification techniques that endow NPs with properties such as immune-system evasion or increased drug-delivery efficiency. These methods are considered as providing “tools” to NPs to gain advantages in various situations. On a more fundamental side, a simple change in the NPs’ geometry (size and shape) can also alter the properties of NPs without the use of any “tools.” For example, the cellular uptake pathway of NPs was demonstrated to be size-dependent, which is a key factor when determining the degree of cytotoxicity (Sun et al., 2016; Sanità et al., 2020).

In another example, the interactions between NPs and cells were investigated with NPs of different shapes. Rod-shaped NPs contain more accessible binding sites than spherical NPs, which promote NP-cell interactions (Salatin et al., 2015; Choo et al., 2021). Therefore, it is important to have a complete understanding of the geometry dependency of NPs so that more comprehensive knowledge can be applied when designing them. The following sections summarize details regarding how geometries of NPs affect the level of internalization as well as cellular uptake pathways.

#### 2.3.1 Size

The size of NPs is an important factor that determines the interaction mechanisms between NPs and cell membranes. For NPs to successfully deliver their cargo to a target cell, it is crucial for the cell to internalize the NPs via endocytosis. To trigger this process, the receptors on the target cells have to be activated by specific ligands on the NPs for the initiation of membrane wrapping. Therefore, increasing the number of ligands on the NP surfaces will promote endocytosis.

To accommodate the increase in the number of ligands, the size of NPs naturally needs to be increased. However, it was shown that for NPs with a diameter greater than 60 nm, steric hindrance and receptor saturation tend to occur, while NPs with a diameter less than 30 nm are unable to drive the membrane-wrapping process. In most *in vitro* studies, the optimum range for cell uptake is 10–60 nm, regardless of the NP core and surface charge (Hoshyar et al., 2016). To elucidate the size effect on cell internalization, many studies have reported various results that conflict with each other. For instance, there are reports indicating that the cellular internalization of functionalized gold NPs varies inversely with size (Elbakry et al., 2012; Wong and Wright, 2016), while other reports state that gold NPs of 50 nm tend to undergo cellular internalization at a higher frequency than gold NPs with smaller size (Liu et al., 2013). The reasons for the variation in results may be the use of different decorating ligands as well as the method of preparation of the gold NPs.

Other than cellular uptake, the size dependency of NPs was also observed in a study of biodistribution (Chen et al., 2015). One should note that a more complicated experimental model should be used in future studies of the size dependency of NPs. It was shown that the effects of multiple parameters are entangled, and it is not possible to use hierarchical cluster analysis (HCA) to define the dependence of biological effects on individual physicochemical properties (Xu et al., 2018). A possible direction for future studies regarding the size dependency of NPs may involve the use of different types of NPs (silver NPs or iron oxide NPs) to build larger model libraries (Alkilany et al., 2013), as well as introducing more parameters into HCA so that a more comprehensive data model may be used for better classifications and predictions.

A final word of advice when dealing with the size of NPs is that it changes when subjected to a biological environment. As mentioned earlier, the tendency to form a protein corona is one of the reasons that may distort the size of the NPs. This complication causes difficulties in size-dependent studies *in vivo* (Sabourian et al., 2020). Any analysis or evaluation should be done while keeping in mind the size of NPs before and after they are introduced into a biological medium (Li et al., 2019).

#### 2.3.2 Shape

In many experiments, NPs have been fabricated in various shapes (most commonly spheres). Other shapes such as rods, triangles, stars, and wires can also be found (Kuo et al., 2007; Millstone et al., 2009; Charan et al., 2012; Shiohara et al., 2015). As mentioned earlier, the interactions between NPs and cells are dependent on the geometric shape of the NPs (Salatin et al., 2015). When comparing rod-shaped NPs with spherical NPs, the cellular uptake of rod-shaped NPs has higher efficiency due to the larger aspect ratio (AR)—i.e., the length-to-width ratio (Huang et al., 2010). Investigations regarding the shape effect of NPs have pushed on further *in vivo* studies. The result of the *in vivo* biodistribution and clearance indicated that NPs of different ARs have a very different fate. For example, NPs with larger ARs tended to accumulate in the spleen, while NPs with smaller ARs were likely to be trapped in the liver of mice after intravenous injection (Huang et al., 2011).

In another example, when spherical MSNs and rod-shaped MSNs were administered orally to mice, rod-shaped MSNs attained higher content in all organs than spherical MSNs. This could be the result of prolonged retention of rod-shaped MSNs in RES organs (the liver and spleen) and the ability to prevent macrophage engulfment, thus increasing their half-life in blood circulation (Zhao et al., 2017c). The effect of shape on the cellular uptake pathway was also investigated by comparing gold NPs of three different shapes: stars, rods, and triangles. It was shown that the endocytosis pathway was strongly associated with the shape of NPs and should be further studied to provide a more detailed mechanism (Xie et al., 2017).

Most reports have pointed out the phenomenon of increased cellular uptake efficiency for large AR NPs, but one should also consider the time required for the cells to wrap themselves around NPs with large AR. It was demonstrated that the internalization rate for rod-shaped NPs was slower than that of spherical NPs (Verma and Stellacci, 2010). The benefits of manipulating the shape of NPs should be evaluated thoroughly before designing NPs while bearing in mind the goals to achieve in a specific biomedical application.

## 3 Future perspectives for NP design

In this review, we have presented various design principles of bioinspired interfaces used in biomedical applications, such as drug delivery, cancer treatment/detection, imaging techniques, and therapeutics. The scope of this review is limited to the development of NP-related techniques, which include derivatives of biological entities comprising the use of various mammalian cell membranes, bacterial membranes, and virus-derived entities. The functionalization of NPs using surface modification was also discussed from the use of common polymers such as PEG to the use of hybrid zwitterions with dual stimuli-responsive capabilities. Last but not least, the effects of geometric properties of NPs were briefly mentioned with the impacts that each property has on the NPs.

The emergence of hybrid membrane-coated NPs in recent year have prompted a new route of development toward the improvement of MNPs. The combination of different cell membrane coatings endows the NP with multiple integrated functions which enables a broader range of applications using a single NP (Zeng et al., 2022; Zhu et al., 2022). However, the complication of the membrane structure may result in an even more complex preparation procedure. Nonetheless, the continuous efforts of the research community will steadily march towards further development.

Nanozyme is also another area of interest that is gaining popularity in recent years due to its unique enzyme-mimetic activities that serve as a highly versatile candidate for many disease therapy (Zhang et al., 2021; Ren et al., 2022). For example, the nanozyme developed by Yang et al. display promising results for the treatment of sepsis (Yang et al., 2022). Surface modifications of nanozyme were also shown to provide anti-tumor properties (Tang et al., 2021). More examples of nanozyme and related applications can be found elsewhere (Hong et al., 2022).

NP-related research is still very active, but it seems to be approaching its limits. Considering that most of the results and data stem from experiments that were conducted under controlled conditions, unpredictable results may occur during actual applications (Vega-Vasquez et al., 2020). This may be why very few developed NP-based drug delivery systems have been approved. On the other hand, recent development in the field of nanodevices has brought about interesting insights into the nanotechnology community. It was demonstrated that a chip device containing poly (3,4-ethylenedioxythiophene)-based nanofiber mats was able to capture/release rare circulating tumor cells by exploiting the nature of the nanomaterial using electrical triggers (Yu et al., 2017). The vast application of nanodevices can also be seen in several works that involve noninvasive prenatal diagnostics (Hou et al., 2017), integration with NPs for a versatile biosensor (Chou et al., 2019), the combination of microfluidic chips for cancer cell isolation (Yu et al., 2019) and neuron manipulation (Hsiao et al., 2011; Hsiao et al., 2016; Tsai et al., 2019). The advancements in the field of nanodevices may play an important role in the future development of nanotechnology. Bearing this in mind, the scientific community should aim toward the integration of current NP technology with different fields of science so as to transform theory into real-life applications.
